# Embodied empathy in translation studies: enhancing global readers' cognitive and emotional engagement with translations of traditional Chinese medicine terminology

**DOI:** 10.3389/fpsyg.2025.1618531

**Published:** 2025-08-15

**Authors:** Tong Zhou, Jinghui Wang

**Affiliations:** Department of Foreign Languages and Literatures, Tsinghua University, Beijing, China

**Keywords:** embodied language comprehension, embodied empathy, traditional Chinese medicine, translation studies, cross-cultural communication

## 1 Introduction

The embodied nature of language comprehension has gained increasing theoretical and empirical support in the fields of linguistics, cognitive science and psycholinguistics (Gallese, 2018; Shtyrov et al., 2023; Garello et al., 2024; Visani et al., 2025). Embodied language comprehension theory holds that language is not a mere abstract symbol system, but is deeply rooted in the sensorimotor and affective experiences of individuals (Niedenthal, 2007; Zwaan and Taylor, 2006). As Gibbs (2005) points out, whereas traditional beliefs tend to view meaning as an abstract entity divorced from bodily experience, embodiment theory emphasizes that the process of language comprehension is frequently accompanied by a bodily simulation of the motor, perceptual, and affective states embedded in linguistic content (Barsalou, 1999; Gallese and Lakoff, 2005; Glenberg and Kaschak, 2002; Hauk et al., 2004). Embodiment is particularly crucial in cross-cultural contexts, as language is not only a medium for information transfer, but also a carrier of cultural and experiential worldviews.

Within this theoretical framework, cross-cultural discourse provides unique challenges and opportunities for embodied language processing research. Traditional Chinese medicine (TCM) discourse is a typical example of this, with a language system that highly integrates bodily metaphors, physiological imagery, and culturally embedded affective models (Pritzker and Hui, 2014; Unschuld, 1989; Wang and Chen, 2023). While conveying medical knowledge, TCM expressions encode an embodied worldview that intertwines physical health and emotional states (Tiquia, 2011). As TCM does not dichotomize mind and body, but views emotions as a unity of physicality and experience, its texts provide rich resources for exploring how readers mobilize embodied meanings in cross-cultural contexts.

Several international organizations have played a pivotal role in the process of standardizing TCM terminology by issuing translation guidelines and reference documents. The World Health Organization (WHO), the International Organization for Standardization (ISO), and the World Federation of Chinese Medicine Societies (WFCMS) have developed translation frameworks for core TCM terminology (Han et al., 2025). Nevertheless, despite relatively coordinated efforts, inconsistencies in terminology translation still exist and a universally recognized uniform standard has not yet been formed (Zhou et al., 2023). Inconsistency in the use of terminology hinders the reader's understanding of the translated text and his or her effective grasp of the cultural meaning and deeper connotations carried by TCM terminology (Ye and Zhang, 2017).

In addition to the problem of terminological inconsistency, the presentation of the translation itself constitutes another obstacle. Many TCM terms tend to be overly abstract and academic in the English translation process, thus weakening their embodied and experiential dimensions. For global readers, whose bodily experiences and cultural frameworks often differ from those of TCM contexts, understanding such expressions requires linguistic decoding along with the simulation of unfamiliar bodily states and emotional experiences. Nevertheless, the disconnection between language, culture, and bodily experience often hinders the successful realization of this simulation process (Beard, 2001; Overgaard, 2017; Rizzolatti and Sinigaglia, 2020).

Against the above background, this paper proposes the concept of embodied empathy as an innovative theoretical perspective for exploring the reception effect of cross-cultural translation. Specifically, this paper explores how translators can, through linguistic choices in translating TCM texts, stimulate readers' sensory arousal and emotional resonance, thus engaging their embodied cognitive resources. The target readership of this paper is a global audience of non-Chinese-speaking backgrounds who are exposed to TCM mainly through translations and lack direct experience and familiarity with its linguistic, cultural and embodied underpinnings. A group of particular interest is the general public readers, those who are more inclined to connect emotionally with the vivid and experiential dimensions of the language than the professional TCM practitioners who value terminological equivalence and clinical precision. Accordingly, the embodied empathy framework and translation strategies proposed in this paper are primarily intended for intercultural communication and public engagement contexts, rather than for clinical, regulatory, or professional translation purposes. While this paper principally develops a conceptual framework and proposes translation strategies based on theoretical analysis, it does not include empirical validation of the embodied empathy model. The aim is to offer a hypothesis-generating perspective on how embodied mechanisms may inform TCM translation. Future research is needed to empirically examine the effectiveness of these strategies, for example, through reader response studies, cognitive-affective testing, or neurocognitive experiments.

## 2 Theoretical foundations

### 2.1 Embodied language comprehension

Recent research in cognitive linguistics and neuroscience emphasizes that language comprehension is not based on purely abstract symbolic representations, but is deeply rooted in sensorimotor and affective systems. Pulvermüller (2018) introduces the concept of Action Perception Circuits (APCs), suggesting that language representations and bodily experiences are integrated at the neural level through such networks and this is supported by Hauk et al. (2004), who show that reading action verbs activates motor cortical areas associated with the corresponding body parts, revealing the somatotopic organization of semantic meaning.

Gallese and Cuccio (2015) build on the theory of embodied simulation by suggesting that language comprehension involves mirroring mechanisms, that is, the simulation of bodily movements and emotional states through pre-reflective neural processes. Their notion of “paradigmatic knowledge” highlights that language users mobilize internalized motor patterns when comprehending linguistic meaning, even in the absence of actual movement. From an enactivist perspective, van Elk et al. (2010) argue that comprehension is not based on representational cognitive structures, but rather is generated dynamically through embodied interactions with the environment, focusing more on procedural than declarative knowledge.

Even in the comprehension of abstract concepts, embodiment still plays an important role. Borghi et al. (2017) propose a “multiple representation view,” which integrates sensorimotor simulation with metaphorical, emotional and social contexts. Overall, these perspectives collectively point to a dynamic view of language comprehension as an emotionally and perceptually based, socially embedded, and interactively generated process, a cognitive schema that also provides theoretical underpinnings for the notion of embodied empathy in translation (Zwaan et al., 2002; Glenberg, 1997; Kaschak et al., 2005; Shiang et al., 2024; Kiefer and Pulvermüller, 2012).

### 2.2 Empathy: cognitive and affective dimensions

Within an embodied cognition framework, empathy is increasingly viewed as a sensorimotor-affective process that integrates perspective-taking and emotional resonance. Psychological and psychotherapeutic studies suggest that empathy is a multidimensional construct, comprising cognitive and affective components that are interrelated yet remain relatively independent (Gladstein, 1983; Goldstein and Michaels, 2021; Hoffman, 1977; Strayer, 1987; Basch, 1983; Bohart and Greenberg, 1997). In this context, cognitive empathy refers to an individual's ability to adopt or infer another's perspectives, thoughts, and affective states; whereas affective empathy involves affective resonance or direct experience of another's emotions (Gladstein, 1983). This dual model lays the groundwork for more embodied empathy research, which seeks to integrate the two empathic mechanisms through a sensorimotor and affective simulation lens.

Simulation theory suggests that empathy arises from the internal reproduction of another person's state, and that its neural mechanisms are mediated primarily by the mirror neuron system (Gallese, 2003). In his model of “shared multimodal space,” Gallese (2003) states that observing another person's actions or emotions activates the observer's own neural circuits, which resonate at the body level. At the level of language comprehension, Gallese and Cuccio (2015) further propose a tripartite model, in which bodily action, affective states, and linguistic structures work together to shape the process of comprehension. The reader generates analogical empathy through sensory movements and affective activation in reading, and enters the linguistic world through a perceptual experience. This view is echoed in the psychological and psychotherapeutic traditions. Rogers (1980) emphasizes that empathy encompasses cognitive insight as well as affective attunement, allowing one to experience another person's world as if it were one's own. Barrett-Lennard (1993) points out that the validity of such affective resonance depends on its successful intersubjective communication. In clinical practice, Cooper (2001) identifies three forms of embodied empathy: allowing the body's resonance to unfold naturally, interpreting bodily cues, and maintaining awareness of one's own embodied state. Nonetheless, he also cautions that empathy is always proximate, and that its alignment is fleeting but influential.

Viewed collectively, these perspectives underscore the multidimensional nature of empathy, in which bodily simulation, emotional resonance, perspective taking and intersubjective communication are intertwined. In language comprehension and translation, particularly with culturally embodied texts like TCM, embodied empathy bridges the gap between semantic access and affective simulation, enabling meaning to emerge through cognitive understanding and affective engagement.

## 3 Embodied empathy framework in TCM translation: conceptual development, embodiment challenges, and translation strategies

### 3.1 Developing an embodied empathy framework for translation studies

Integrating insights from embodied language comprehension and empathy theory, this paper proposes a novel theoretical framework, embodied empathy, for translation studies, shedding light on how experiential meaning can be conveyed across linguistic and cultural boundaries. Embodied empathy, as conceptualized in this paper, refers to the target reader's capacity to simulate the bodily sensations, emotional state and cultural logic embedded in the source language expression. Its realization depends on the translator's effective mobilization of the reader's sensorimotor system and emotional schema by the translated text, so that the comprehension process transcends the purely cognitive level and finally achieves an embodied experience of meaning. Operationally, this concept can be indicated by the degree to which a translation evokes sensory imagery, emotional resonance, and culturally grounded associations in the reader's experience. Such effects may be preliminarily assessed through reader-response studies, experiential feedback, or cognitive-affective measures that examine whether the translation stimulates embodied simulation beyond mere semantic comprehension.

Within this framework, the success of translation depends not only on semantic equivalence, but also on experiential equivalence as reflected in the reader's embodied engagement with the source text (Alexieva, 2018). A number of central questions need to be carefully considered in the translation process: Can readers from global biomedical backgrounds simulate the bodily-emotional states when reading TCM translations? Are their pre-existing embodied experiences sufficient to support the corresponding emotional resonance? If not, how can the translator intervene to bridge this experiential gap? In this sense, the translator functions as an intertextual interpreter and a cross-modal mediator bridging embodied worlds. His or her task is to reconstruct a perceptual-affective ecosystem in the target language, so that it retains the physiological realism, sensory vividness and emotional tension of the source text. This requirement is particularly paramount in the translation of TCM, because the bodily processes described in TCM are deeply rooted in cosmological and cultural metaphors that often lack direct counterparts in global epistemologies.

To operationalize the theoretical framework, this paper defines three interrelated dimensions: sensorimotor simulation, affective resonance and cultural mapping. These three dimensions work synergistically to facilitate readers' cognitive understanding of the translated content as well as their deeper engagement at the physical and emotional levels. In highly culturally saturated domains such as TCM, embodied empathy provides a crucial theoretical reference for assessing whether translations are able to achieve a balance between semantic equivalence and experiential depth.

Sensorimotor simulation refers to the ability of readers to simulate bodily actions or perceptual experiences (e.g., movement, temperature, texture, etc.) on a mental level during language comprehension. This simulation does not involve actual bodily movements, but is achieved by the activation of the corresponding sensorimotor pathways in the brain (Pulvermüller, 2018; Hauk et al., 2004). For instance, when a reader reads the expression “a chilling gust of wind,” the reader not only grasps the semantic content of “cold,” but may also internally recreate the tactile sensation of the cold wind on the skin. If the translation does not sufficiently evoke similar sensory associations in the target readers, the embodied experience may only be partially activated, even if the semantic rendering is equivalent.

Affective resonance enables readers to emotionally attune to the tone of a text, comprehend its affective connotations, and ultimately experience the emotional state evoked by the language (Gallese, 2003; Rogers, 1980; Cooper, 2001). For example, the expression “a fire rising in the chest with nowhere to escape” not only conveys the abstract emotional concept of anger, but also evokes an embodied emotional intensity that allows the reader to feel the agitation rising within. Translations that effectively convey emotional content and stimulate readers' emotional involvement are more likely to generate deeper empathy among the readers of the translated language.

Cultural mapping is defined as the ability of readers to project and interpret culture-specific physical and emotional metaphors within their own conceptual framework. This process determines whether the translation can effectively fit into the reader's cultural-cognitive schema, thus enabling the resonance of meaning and experience (Borghi et al., 2017). In TCM, for example, expressions such as “ascending of liver qi pattern” [World Health Organization (WHO), 2022] draw upon cosmological and metaphorical systems rooted in Yin-yang and Five-elements theory. Here, the term “liver” is not merely an anatomical reference, but a culturally coded symbol of emotional regulation and systemic flow. Translators need to consider how to reconstruct these embodied metaphors so that target readers can build conceptual bridges to the source culture's embodied worldview.

Though sensorimotor simulation, affective resonance, and cultural mapping can be analytically distinguished as three operational dimensions, they function interactively in actual discourse processing. Embodied empathy is not a simple sum of these components, but an emergent phenomenon arising from their dynamic and mutually reinforcing interplay. As such, embodied empathy serves as a conceptual bridge linking the embodied-cognitive mechanisms of language comprehension with the cross-cultural dynamics of translation reception. It foregrounds the affective and perceptual fidelity of translation, inviting target readers to intellectually comprehend and emotionally engage with the embodied meanings of the text. Such a perspective pushes translation studies toward a more integrated mode of understanding, in which language, body and culture are seen as an inseparable organic whole in the process of meaning construction.

To ground this conceptual framework in actual translation practice, this paper draws upon representative examples from the *WHO International Standard Terminologies on Traditional Chinese Medicine* (2022). Issued by the World Health Organization, this guideline provides standardized English translations for key TCM terms across three major domains: fundamentals of traditional Chinese medicine; diagnosis, patterns, and constitution; and treatment principles, methods, and therapies. These terminologies offer a valuable corpus for analyzing how embodied meanings are retained, transformed, or diminished in cross-cultural translation. In the following sections, selected examples are used to illustrate how challenges related to sensorimotor simulation, affective resonance, and cultural mapping manifest in translation, and how corresponding strategies, guided by the embodied empathy framework, are proposed in response.

### 3.2 Embodiment-based challenges in traditional Chinese medicine translations

On the basis of the theoretical model of embodied empathy, this paper further outlines three main types of challenges in cross-cultural translation, corresponding to the aforementioned operational dimensions: sensorimotor simulation, affective resonance, and cultural mapping. These challenges are particularly prominent in the translation of TCM, as the language is highly intertwined with bodily experience, emotional logic, and cosmological worldview (Hsiao et al., 2008). It should be emphasized that these three types of challenges are not independent of each other, but often show complex interactions and overlapping relationships in the actual translation process.

#### 3.2.1 Sensorimotor attenuation

The first challenge lies in that the translation may only partially activate the target reader's sensorimotor system, which can lead to a reduced sense of bodily vividness compared to the original. TCM language frequently encodes embodied states, such as cold, heat, pressure, and flow, through rich, image-based metaphors. Yet in translation, conceptual clarity often takes precedence over sensory immediacy, and the translators often render vivid, somatically charged language into abstract biomedical terms (Ye and Zhang, 2017). As sensorimotor grounding is central to embodied comprehension (Pulvermüller, 2018), any loss in sensory imagery can limit the reader's cognitive-affective immersion in the translated text.

For example, the expression 寒湿困脾证 (hán shī; kùn pí zhèng), standardized as “cold dampness affecting the spleen pattern” in the WHO TCM terminology guideline, exemplifies a significant case of sensorimotor attenuation in translation [World Health Organization (WHO), 2022]. In the source term, 寒 (hán, “cold”) and 湿 (shī;, “dampness”) are not abstract meteorological descriptors but directly invoke tactile and thermal experiences: cold connotes chill, stiffness, and contraction; damp suggests heaviness, stickiness, and stagnation. The character 困 (kùn, “affect”) further reinforces this embodied image by implying functional sluggishness and visceral inertia, capturing a bodily state of internal blockage.

However, the English translation, while semantically equivalent, tends to render these vividly embodied sensations in more technical biomedical terms. The structure “cold dampness affecting the spleen pattern” adopts a clinical and formulaic register, which may reduce the tactile immediacy and perceptual intensity present in the original. In particular, the somatic weight of 困 (kùn, “affect”), a key experiential cue, becomes less salient, potentially leading to a flattened sensorimotor profile.

This example highlights a central concern in the embodied empathy framework: when the translation has limited capacity to activate the target reader's sensorimotor system, the resulting text may remain intelligible but offer reduced experiential engagement. Such attenuation undermines the capacity of translation to convey the perceptual and bodily dimensions essential to embodied understanding.

#### 3.2.2 Affective disconnection

The second challenge involves the disruption of emotional resonance in translation. Numerous TCM expressions convey emotional experience through embodied metaphors that reflect intertwined physiological and affective states, such as frustration, repressed anger, or anxiety (Brehaut et al., 2007). However, when these expressions are rendered into emotionally neutral or clinically detached formulations, the original affective intensity is significantly diminished. As a result, the target-language reader may cognitively grasp the semantic content but is unlikely to engage emotionally with the experience described. As empathy theory suggests (Gallese, 2003; Rogers, 1980), affective resonance is not merely ornamental; it constitutes a foundational mechanism for empathic understanding. When the emotional tenor of the original is flattened, the pathways for embodied empathy are obstructed, limiting the translation's capacity to foster experiential engagement.

The expression 肝火上炎证 (gān huǒ shàng yán zhèng), translated by the World Health Organization in 2022 as “upward flaming of liver fire pattern,” exemplifies a highly embodied and emotionally charged linguistic construct in TCM. It encapsulates a deep entwinement of somatic perception, emotional experience, and culturally embedded metaphor. In TCM discourse, the liver is not merely a physiological organ but a symbolic and functional center of emotional regulation, particularly associated with the emotion of anger. The canonical principle of the liver reflects this culturally coded mind-body integration, where internal emotional turbulence directly affects physiological processes. The term 火(huǒ, “fire”) functions dually as a pathological agent and a metaphorical representation of affective arousal, signaling sensations of heat, agitation, and impulsivity. Meanwhile, 上炎 (shàng yán, “upward flaming”) introduces directional and kinetic imagery, simulating an embodied experience of internal energy surging upward during emotional outbursts.

By contrast, the standardized English translation, “upward flaming of liver fire pattern,” as adopted in the WHO TCM terminology guideline, while terminologically equivalent, tends to attenuate the affective intensity conveyed in the original. Although flaming nominally retains the imagery of fire, its metaphorical resonance in English appears relatively diffuse and may not strongly evoke stable emotional associations. The phrase “liver fire pattern” adopts a clinicalized structure that places emphasis on biomedical taxonomy, which may limit the experiential richness embedded in the source term. Consequently, the translated expression functions more as a diagnostic label, with reduced physiological and emotional immediacy compared to the original. The dynamic force, directional intensity, and emotional agitation present in the Chinese expression are correspondingly diminished. When affective schemas related to anger or heat are not fully engaged, the potential for activating embodied empathy becomes considerably constrained.

This case illustrates the challenge of affective disconnection, a breakdown in the affective resonance process described in the proposed theoretical framework. Despite semantic equivalence, the translation may only partially elicit embodied emotional engagement, leaving readers intellectually informed while generating attenuated affective resonance. As a consequence, the mechanism of embodied empathy remains less fully activated.

#### 3.2.3 Cultural incommensurability

The third challenge stems from fundamental differences between the cultural models of body and health embedded in TCM and those assumed by global biomedical readers. Unlike the Western anatomical framework, TCM conceptualizes organs such as the liver and spleen as part of a dynamic system governed by qi and yin-yang balance (Runhu, 2015; Zhao et al., 2023). These expressions are rooted in cosmological and metaphorical assumptions that lack direct counterparts in Western epistemologies. Consequently, even precise lexical renderings may fall short in eliciting embodied understanding if the reader lacks the necessary cultural-cognitive schema, ultimately disturbing the alignment between language, body, and culture.

Consider the term 命门之水 (mìng mén zhī; shuǐ), rendered in the WHO TCM terminology guideline as “the water of the gate of life.” This expression exemplifies a culturally and somatically embedded TCM construct whose meaning extends far beyond its surface lexicon. According to the interpretations provided by both classical texts and contemporary scholarship, 命门 (mìng mén, “gate of life”) denotes not merely an anatomical site located between the kidneys, but rather serves as a vital energetic hub associated with reproductive function, metabolic regulation, and life force (Li, 2005; Meng, 2006; Chen and Zheng, 2013). 水(shuǐ, “water”) refers to stored yin essence, conceptually balancing the yang fire, and representing inner cooling, moistening, and sustaining forces. From the perspective of embodied cognition, the phrase encodes sensations of thermal regulation and vital flow, reflecting a worldview in which physiological and cosmological orders are intertwined.

While the WHO translation maintains a close literal correspondence, it may not fully convey the cultural and embodied dimensions embedded in the original. In English, the “gate of life” does not readily evoke a familiar reference to an organ or energy center, which may render the term less accessible or metaphorically resonant. Similarly, the term “water” has limited affinity with the concepts of “yin vitality” and “essence” in TCM concepts. Despite the lexical equivalence of this translation, its ability to activate the cultural-bodily schema of the target readers is relatively limited, and the concept may remain abstract, making it difficult to realize the in-depth communication of embodied experience.

This case exemplifies the challenge of cultural incommensurability in translation, where the worldview encoded in the source language terminology is difficult to be mapped perceptually or emotionally onto the target language culture. The result constitutes a state of semantic suspension: although the translation achieves equivalence at the lexical level, it may not comprehensively stimulate embodied or affective experiences, thereby limiting the possibility of deeper intercultural understanding.

Taken together, the three types of challenges, namely sensorimotor attenuation, affective disconnection and cultural incommensurability, constitute the most prominent embodied barriers in cross-cultural translation of TCM. As a typological framework, it reveals the key disruptions in embodied empathy and provides a diagnostic tool for assessing whether the translation goes beyond basic semantic equivalence and realizes a higher level of translation adequacy.

### 3.3 Translation strategies informed by embodied empathy

From the perspective of embodied empathy, translation is not merely a matter of semantic transfer, but a reconstructive act that seeks to preserve and reawaken the source text's perceptual, somatic, and affective dimensions (Robinson, 2014; Chávez, 2009). In response to the preceding analysis of the three embodiment-based challenges, the following section introduces three corresponding translation strategies developed in this paper to address each of these barriers. This paper primarily develops its strategies for intercultural communication targeting non-specialist global readers, aiming to enhance experiential engagement and cultural resonance. While the framework may offer insights for professional translation practice, its main focus is not on terminological adaptation for clinical or regulatory purposes.

#### 3.3.1 Sensory activation

To mitigate the effects of sensorimotor attenuation, this paper introduces the strategy of sensory activation. This approach emphasizes the restoration of perceptual immediacy and bodily vividness in translation by foregrounding sensory metaphors, tactile descriptors, and experiential analogies. Instead of relying solely on technical formulations, the translator can reconstruct the source text's somatic imagery, which helps re-engage the reader's sensorimotor pathways and strengthen embodied empathy.

In this paper, the standardized WHO translation of 寒湿困脾证 (hán shī; kùn pí zhèng) as “cold dampness affecting the spleen pattern” is rendered as “a condition of internal heaviness and chill that obstructs the spleen's activity,” which exemplifies the sensory activation strategy within the embodied empathy framework. Compared to more abstract biomedical formulations, this rendering appears to offer greater potential for sensorimotor simulation, as it seeks to preserve the tactile and kinesthetic dimensions embedded in the source term. The phrase “internal heaviness and chill” evokes direct bodily imagery, allowing target readers to mentally simulate sensations of cold, weight, and stagnation. Additionally, the verb “obstructs” introduces a dynamic element of physiological resistance, reflecting the somatic inertia implied by 困(kùn, “obstruct”). Hence, this translation facilitates an embodied mode of comprehension, reinforcing the experiential and empathetic connection between reader and text.

Rather than focusing solely on semantic equivalence, this strategy aims to enhance sensory immediacy and bodily vividness by reintroducing concrete perceptual cues and kinetic imagery, elements that may be attenuated in the process of terminological standardization. In counteracting the abstraction typical of biomedical renderings, the approach enables readers to simulate the somatic experiences encoded in the source term. This, in turn, enhances the translation's potential to evoke embodied empathy and maintain the perceptual-affective integrity of the original TCM expression.

#### 3.3.2 Affective reenactment

In light of the challenge of affective disconnection in translation, this paper advances the strategy of affective reenactment, which aims to reactivate the emotional dynamics embedded in the source text by reconstructing its affective imagery and emotional trajectory. The goal is to preserve the perceptual-affective interplay encoded in TCM terminology while enhancing the experiential depth and empathetic efficacy of cross-cultural translations.

When the source term contains implicit emotional triggers, such as anger or worry, but the target language lacks corresponding body-emotion mappings, translators may render the causal logic of emotional arousal more explicit. This can guide target readers in understanding the affective motivations underlying physiological expressions. Additionally, affective reenactment involves amplifying and concretizing the somatic imagery, directionality, and dynamic movement inherent in the original expression, while favoring emotionally potent and kinetically charged verbs over neutral technical formulations, thereby enhancing embodied expressivity.

An illustrative example is the WHO's standardized translation of 肝火上炎证 (gān huǒ shàng yán zhèng) as “upward flaming of liver fire pattern.” While this version maintains terminological equivalence, it may somewhat reduce the emotional intensity conveyed in the original. In contrast, the alternative translation proposed in this paper, “a surge of blazing heat rising from the liver in response to unexpressed anger,” demonstrates the affective reenactment strategy. It retains the thermal and directional aspects of 上炎 (shàng yán, “upward flaming”), explicitly articulating the underlying emotional causality linked to anger (“in response to unexpressed anger”). The word “surge” evokes a sudden, forceful upward motion, and the adjective “blazing” triggers vivid somatic sensations, jointly enriching the embodied dimension of the expression.

Through the reconstruction of the connection between emotion, body, and cultural metaphor, this approach engages the reader's affective repertoires. Instead of leaving the reader emotionally detached, the translation activates the internal dynamics of emotional tension embedded in the source, reviving its expressive force. In this way, affective reenactment acts both as a remedy for potential emotional flattening and a channel for restoring the experiential depth essential to embodied empathy.

#### 3.3.3 Cultural grounding

Anchored in the theoretical framework of embodied empathy, cultural grounding seeks to bridge the interpretive gap caused by the target culture's lack of embodied background knowledge. It does so through a dual mechanism: explicitation and the construction of cultural-bodily schemas. Cultural grounding aims, for one thing, to help target-language readers establish cognitive footholds within their own cultural frameworks, and for another, to re-embody abstract or symbolically dense TCM expressions in ways that evoke sensory and cultural associations.

More specifically, when source-language terms lack perceptible cultural or bodily referents in the target culture and may not readily engage readers' sensorimotor or conceptual schemas, translators are encouraged to actively reconstruct an embodied understanding. This may involve cultural explanation, analogical reasoning based on bodily functions, or symbolic cues, allowing readers to simulate the philosophical and physiological worldview embedded in the source expression through their own experiential frameworks.

To implement the cultural grounding strategy, this paper proposes a composite approach termed cultural-embodied explication, which integrates two complementary methods. First, cultural mapping compensation addresses the absence of equivalent philosophical or somatic frameworks in the target culture by introducing analogous concepts (e.g., energy flow, yin-yang balance, meditative vitality) that provide accessible interpretive bridges. Second, metaphorical analogy and contextual elaboration entail supplementing the translation with metaphorical parallels or brief contextual explanations to reconstruct the cultural and embodied meanings underlying the original expression.

This paper proposes a revised translation of 命门之水 (mìng mén zhī; shuǐ) as “the yin essence stored at the life-gate, a vital reservoir of cooling energy that balances the body's inner fire.” Unlike the WHO's literal translation “the water of the gate of life,” which maintains lexical equivalence but may not comprehensively engage the target reader's cultural-bodily schema, the revised version employs the strategy of cultural grounding through culturally and somatically enriched explication. Specifically, the term “yin essence” explicitly introduces the somatic and cosmological dimension of 水 (shuǐ, “water”), which in TCM refers not to physical water but to an abstract, nourishing, cooling substance essential for sustaining life. The phrase “stored at the life-gate” locates 命门 (mìng mén) as a symbolic and functional center rather than an anatomical site, while the elaboration “a vital reservoir of cooling energy that balances the body's inner fire” draws on familiar metaphors of energy balance and inner equilibrium to evoke the yin-yang dynamic.

This contextual elaboration helps readers simulate the embodied sensations of thermal regulation and energetic flow, making the expression experientially intelligible. Instead of merely conveying lexical meaning, the translation incorporates cultural mapping compensation (e.g., analogies to energy centers or balance models in holistic health) and contextual elaboration (e.g., expressions such as “yin essence” or “cooling energy”) to anchor the source term in culturally resonant schemas. This multidimensional approach helps to restore the vividness and philosophical depth of the terminology, thereby eliminating the semantic suspension and reactivating the interpretative channels necessary for the realization of embodied empathy and cross-cultural understanding.

To summarize, the three proposed strategies of sensory activation, affective reenactment and cultural grounding constitute an organic theoretical framework for addressing the challenges of embodiment in TCM translation. These approaches emphasize sensory immediacy, emotional resonance and culturally embedded bodily schema, transcending the singular pursuit of semantic equivalence. Such a shift transforms translation into a medium of cross-cultural embodiment, enabling readers to intellectually comprehend and viscerally engage with the conceptual and philosophical logic of TCM.

## 4 Embodied empathy framework in TCM translation: theoretical contributions, practical implications, and scope limitations

The theoretical innovation of this paper lies in extending the concept of embodiment from cognitive linguistics into the field of translation studies, with a particular focus on the cross-cultural translation of TCM terminology. Centered on the tripartite coupling of language, body, and culture, the proposed framework of embodied empathy in translation offers an operational model that moves beyond conventional applications of embodiment, typically limited to everyday verbs or sensory expressions, and demonstrates its relevance in highly abstract and culturally embedded professional discourse. While sharing the common objective of enhancing the accuracy and effectiveness of TCM translation, this paper seeks to contribute to ongoing efforts by identifying specific areas where embodied mechanisms may enrich cross-cultural comprehensibility. This paper fills a critical gap in current translation studies by uncovering the somatic schema and experiential underpinnings embedded in even the most technical TCM terminology, which in turn opens up a novel pathway for reconstructing experiential depth and emotional expressivity in cross-cultural medical discourse.

The findings of this paper resonate with and extend several key arguments in the field of TCM translation. Notably, Li's (1996, 2008) advocacy of the principle of ethnic specificity underscores the epistemological incommensurability between Chinese and Western medical paradigms, emphasizing the importance of preserving the culturally embedded worldview of TCM terminology. The analysis in this paper echoes the above viewpoints, pointing out that embodied empathy helps translators retain the emotional and bodily dimensions of culturally specific terms in the translation process, thus reinforcing their unique conceptual identities in the target language. Meanwhile, the cultural schema theory mentioned by Zhou and Wang (2013) is further substantiated in this paper. By highlighting the role of body simulation in translation reception, this paper presents the dynamic activation mechanism of cultural knowledge in the process of cross-cultural comprehension from a sensorimotor perspective. Moreover, the reader-oriented approach proposed in this paper complements the emphasis on translators' cognitive differences discussed by Li and Sang (2023), by focusing on how linguistic strategies can help non-Chinese readers simulate unfamiliar embodied experiences. In line with the concept of Yu et al. (2022) of the balanced application of foreignization and domestication strategies, the embodied empathy framework proposed in this paper provides a hybrid path of cultural specificity and acceptability for translation practice. Finally, this paper also responds to Xie's (2000) call for greater international collaboration in the standardization of TCM terminology, providing a new theoretical perspective for Chinese and global readers to reach a shared experiential understanding through translation.

Although the translation strategies proposed in this paper may appear to diverge from established professional norms, or even conflict with, the standardization principles advocated in the *WHO International Standard Terminologies on Traditional Chinese Medicine* [World Health Organization (WHO), 2022], they nonetheless offer significant theoretical and practical value. A close analysis of the guideline's structure reveals a multilayered presentation of terms. The “English term” column usually adopts highly abstract and academic expressions, which often lack embodied resonance and are less likely to stimulate the sensory engagement of the target language readers. In contrast, the “Synonyms” column tends to adopt more vivid expressions with sensory imagery, dynamic metaphors, and affective undertones, reflecting the intention to retain the dimension of embodied meaning in the translation process. Taking “伤寒类病, shāng hán lèi bìng” as an example, the “English term” column translates it as “cold damage,” which is semantically close to the literal meaning, but may not fully capture the cultural specificity or experiential immediacy associated with the term in TCM contexts, especially in reference to the core embodied experience of acute febrile illnesses induced by exogenous cold pathogens. In contrast, the translations provided in the “Synonyms” column, such as “exogenous febrile disorders” and “cold injury,” are more effective in activating readers' cultural schemas and bodily imaginations. Among them, “exogenous febrile disorders” highlights the characteristics of acute fever in exogenous diseases, while “cold injury” draws on people's more intuitive associations of cold as a cause of illness. Similarly, “胃火证, wèi huǒ zhèng” is expanded to “stomach heat exuberance pattern” and “excess stomach heat pattern” in the “Synonyms” column, which more concretely portray the physical characteristics of internal heat exuberance.

The above examples reveal a potential compensatory mechanism commonly observed in terminology systems such as those of WHO, ISO, and WFCMS, which attempt to balance the expressive tensions of embodied language with the technical demands of terminological standardization. Specifically, compilers often include complementary expressions in the “Synonyms” column to enhance both conceptual clarity and perceptual accessibility. This practice suggests that translating TCM's highly symbolic and experiential lexicon requires more than rigid adherence to standard equivalence; it calls for a flexible approach that negotiates between embodied empathy and regulatory consistency. A notable example of this balancing effort is the WHO's standardized term 峻下逐水药 (jùn xià zhú shuǐ yào), rendered as “drastic water-expelling medicines.” Here, “drastic” aptly conveys the forceful purgative effect, while “water-expelling” specifies its function. Yet from the perspective of embodied empathy, this translation remains relatively clinical, potentially falling short of evoking the visceral intensity and dynamic bodily impact, a concept traditionally linked to powerful, aggressive purgation. This example illustrates a partially successful integration of standardization with experiential resonance, while also highlighting opportunities for further enriching TCM translations through the enhancement of sensory and affective dimensions.

Though this paper constructs a theoretical framework for the translation of TCM terminology based on embodied empathy and proposes three corresponding strategies, several limitations remain and warrant further discussion. To begin with, the number of TCM terms analyzed is relatively small, focusing chiefly on a few representative diagnostic expressions. As such, the findings may not fully capture the overall complexity of the TCM terminological system. However, given that the primary objective of this paper is to propose and preliminarily validate the applicability of embodied empathy theory in the context of TCM translation, the use of illustrative cases serves to clearly demonstrate the translation challenges and responsive strategies associated with embodied dimensions. Additionally, TCM comprises a wide range of text types, including classical theoretical texts, clinical guidelines, pharmacopeias, and popular science materials, each with distinct communicative functions and embodied representational features, as well as differing audience expectations (Luo and Deng, 2017). This paper has not yet explored how these variations may necessitate differentiated translation strategies, which will be addressed in future research.

Lastly, this paper principally focuses on intercultural communication targeting general, non-specialist readers, with an emphasis on fostering experiential resonance and perceptual engagement in translation. It does not directly address issues related to terminological adaptation or clinical applicability within professional medical contexts. Nevertheless, it is acknowledged that the practical demands of standardization, especially in clinical or regulatory environments, require translators to carefully navigate the balance between preserving embodied meaning and ensuring terminological consistency. In such contexts, translators may adopt a layered approach: maintaining standardized terminology for core diagnostic or regulatory expressions, while enriching surrounding explanatory content or supplementary materials with embodied, culturally resonant language. This dual strategy allows for both compliance with professional norms and the retention of experiential depth. Introducing embodiment as a complementary evaluative dimension thus enhances existing translation paradigms centered on semantic equivalence and standardization, while also offering insights into the communicative transition between expert discourse and public understanding.

## 5 Conclusion

This paper has proposed an embodied empathy-oriented framework for the cross-cultural translation of TCM terminology, addressing three core challenges: sensorimotor attenuation, affective disconnection, and cultural incommensurability. In response, it introduces three targeted translation strategies, sensory activation, affective reenactment, and cultural grounding, each designed to retain the perceptual, emotional, and culturally situated dimensions of TCM discourse. These strategies aim to move beyond semantic equivalence, advocating for a translation approach that reactivates the embodied experiences encoded in the source text. The restoration of somatic imagery, emotional intensity, and culturally resonant schemas enables the model to foster deeper experiential engagement and improve the target reader's empathetic and cognitive access to TCM concepts.

While this approach may depart from the terminological standardization logic emphasized in institutional guidelines such as the WHO TCM terminology guideline, it reveals the interpretive and experiential gaps that can arise from overly abstract or clinical renderings. In this light, this paper offers both a theoretical extension of embodiment theory into the field of translation studies and a practical response to the growing need for culturally sensitive, experientially rich translations in global TCM communication. It argues for a more dynamic balance between professionalization and perception, between standardization and resonance, providing a foundation upon which future interdisciplinary and reader-oriented research may continue to build.

Although limited in scope and reader context, the findings highlight the importance of balancing technical consistency with embodied intelligibility. Building on this paper, future research may proceed along several fruitful lines. First, empirical validation is needed to assess the effectiveness of embodied empathy in translation reception. This could involve reader response studies, eye-tracking methodologies, or sensory perception experiments to examine how different translation strategies impact cognitive and affective engagement. Moreover, comparative studies across languages and cultures could investigate the diversity of bodily metaphors and perceptual schemas, contributing to a more inclusive and empirically grounded model of linguistic embodiment. Finally, to further enhance the practical applicability and theoretical reach of the current model, future research may benefit from exploring variations in reader profiles, text types, and professional contexts, particularly in medical or clinical translation domains.
